# Supplementary material for: Electrochemical carbon–carbon coupling with enhanced activity and racemate stereoselectivity by microenvironment regulation
Source: Nat Commun. 2023 Oct 30;14:6925. doi: 10.1038/s41467-023-42724-2 (PMC10616095; doi:10.1038/s41467-023-42724-2)
Supplement: Supplementary file 1 — Supplementary Information [file 41467_2023_42724_MOESM1_ESM.pdf]

Supplementary Information for

Electrochemical carbon−carbon coupling with enhanced activity and controlled stereoselectivity by microenvironment regulation

Kejian Kong[1], An-Zhen Li[1], Ye Wang[1], Qiujin Shi[1], Jing Li[1] Kaiyue Ji[1] and Haohong Duan[1, 2, 3]*

[1]Department of Chemistry, Tsinghua University, Beijing, China.
[2]Haihe Laboratory of Sustainable Chemical Transformations, Tianjin, China.
[3]Engineering Research Center of Advanced Rare Earth Materials, (Ministry of Education), Department of Chemistry, Tsinghua University, Beijing, China.

Table of content

Supplementary Notes 1-6

Supplementary Figures 1-37

Supplementary Tables 1-8

Supplementary References 1-23

1. Supplementary Notes

Supplementary Note 1: electric double layer structure with cation adsorption

At a typical electrode–electrolyte interface, most cations cannot penetrate water layer to directly contact with the electrode's interface because of high degree of hydration. Therefore, non-specific adsorption takes place for most cations and an outer Helmholtz plane (OHP) is formed. However, quaternary ammonium cations tend to penetrate the water layer and are directly adsorbed on the electrode's surface, giving rise to the specific adsorption with the formation of inner Helmholtz plane (IHP)[1], as reported for Cs^+, Tl^+ ions with weak hydration shell (which is also confirmed in Supplementary Fig. 36a ,b). Therefore, we rationalize that CTAB is directly adsorbed on the electrode's surface.

Supplementary Note 2: the role of hydrophobic chains in ordered arrangement

For organic molecules with long chain, they intend to spontaneously form ordered molecular membrane through intermolecular force, with the formation of so-called self-assembled monolayers (SAMs)[2]. Therefore, we posit that CTAB molecules would arrange orderly over CP interface under the electrochemical conditions because of the hydrophobic interaction between the hexadecyl groups. To demonstrate this possibility, a series of quaternary ammonium cations without hexadecyl group were selected, including DTAB (with shorter hydrophobic chain), HDBAB (with larger head group), TMAB and TBAB (without hydrophobic chains) for investigation. The modified CP electrodes are denoted as CP-DTAB, CP-HDBAB, CP-TMAB and CP-TBAB.

For CP-DTAB and CP-HDBAB, they display similar trend over CP-CTAB in EIS measurements (Supplementary Fig. 20), suggesting DTAB and HDBAB at CP interface also undergo conformational transformation when more negative potential is applied. However, the associated transition points are more negative than that for CP-CTAB, indicating their arrangement is less ordered compared with CTAB. This is reasonable because the short chain in DTAB shows weaker hydrophobic interaction, and the large head group in HDBAB displays higher steric hindrance. In evident contrast, the transition points are not observed for CP-TMAB and CP-TBAB (Supplementary Fig. 21), suggesting that hydrophobic chains in the quaternary ammonium cations play a pivotal role in potential-dependent conformational transformation.

Supplementary Note 3: identification of rate-determining step

Normal pulse voltammetry (NPV) experiments show that the limiting diffusion current (I_d) over CP or CP-CTAB is not affected by either benzaldehyde concentration or stirring rate in the reaction (Supplementary Fig. 23). In addition, I_d appears at more negative potential (approximately −1.8 V) compared with the reaction potential (−1.2 V~ −1.5 V). These results suggest that C–C coupling over CP and CP-CTAB is not dominated by diffusion or other mass transfer-related processes. Note that CP-CTAB even displays lower I_d value compared with CP (Supplementary Fig. 23), which we tentatively attribute to CTAB adsorption may prevent benzaldehyde molecules from approaching the CP interface at diffusion-controlled region, which in turn mitigates the current density. Hence, we speculate that electrochemical benzaldehyde C–C coupling is mainly controlled by kinetics under the reaction conditions.

The Tafel plots display a slope of 87.1 and 91.3 mV/dec over CP and CP-CTAB electrode, respectively, more than 60 mV/dec (Supplementary Fig. 24), revealing that the RDS of benzaldehyde C–C coupling involves the transfer of the first electron, which presumably follows a proton-coupled electron transfer (PCET) process of benzaldehyde that delivers carbon radical intermediates[3], as evidenced by electron paramagnetic resonance (EPR) (Supplementary Fig. 25). The participation of water in the PCET process is confirmed by kinetic isotope effect (KIE) experiments (Supplementary Fig. 25), which shows that the reaction rate is affected by proton transfer[4]. Based on the determination of RDS, we deduce that CTAB may play a significant role in modulating reaction intermediate, namely ketyl radical, in the PCET process, thereby facilitating the reaction rate.

Supplementary Note 4: site-blocking effect

According to the previous reports, a site-blocking effect would obviously decrease the reaction rate in various electrocatalytic reactions, such as in methanol oxidation[5], oxygen reduction[6], hydrogen oxidation[7] and carbon dioxide reduction[8]. The site-blocking effect is stemming from competitive adsorption of surfactants and reactants, which inhibits reactant transformation. Therefore, site-blocking effect is irrelevant with electrical factors[5]. As a result, although R_{ct} values of the short-chain cations were affected by electrical factors such as local electric field and dipole interaction, the degree of negative deviations from the linear relationship is mainly affected by the intrinsic structural factors of the short-chain cations.

Specifically, the reaction rate is correlated with the size of cation head group (e.g., TMAB < TEAB < TPAB < TBAB, and TMAB < BTAB < TBAB). The negative relationship between the head group size and negative deviation from linear relationship (Fig. 4i) can be attributed to the influence of cations coverage. For adsorption sites with a certain area, cations with decreased size show higher stacking density, and the degree of resistance to the adsorption site would be more intense. For instance, cations with smaller size (such as TMAB) induce higher coverage over CP interface, resisting benzaldehyde from approaching the interface[9].

Taken together, the quaternary ammonium cations display both promoting and inhibiting effects on the reaction rate, which are stemming from the dipolar interaction and site-blocking effect, respectively. For cations with longer chains (such as CTAB), the former effect is prominent and the activity is promoted, while for cations with shorter chains, the latter effect becomes more important and the reaction rate is inhibited.

Supplementary Note 5: activation energy (E_a) and activation entropy (ΔS^*) in transition state theory

When an external potential is applied, the Fermi level of electron is varied because of charge accumulation at electrode–electrolyte interface, which in turn regulates the solvent reorganization energy of reactants, consequently reducing the reaction activation energy. According to Arrhenius equation (1):

$$lnk = -\frac{E_a}{RT} + lnA \tag{1}$$

where k is rate constant, E_a is activation energy, R is gas constant, T is system temperature and A is a preexponential factor related to activation entropy (ΔS^*).

According to transition state theory, the reaction rate is related with the Gibbs free energy change (ΔG^*) between the ground state and the transition state (or called activation state), which can be described with activation enthalpy (ΔH^*) and activation entropy (ΔS^*). Meanwhile, the RDS of electrochemical pinacol coupling involves electron transfer, thereby transition state theory can be leveraged in our discussion on the activity promoted by CTAB modification. As a result, reaction rate constant (k) can be presented as:

$$k = \frac{k_B T}{h} K_c^* = \frac{k_B T}{h} e^{-\frac{\Delta G^*}{RT}} = \frac{k_B T}{h} \cdot e^{\frac{\Delta S^*}{R}} \cdot e^{-\frac{\Delta H^*}{RT}} \tag{2}$$

Meanwhile, the activation energy (E_a) can be presented as follows:

$$E_a = RT^2 \frac{dlnk}{dT} = RT + RT^2 \frac{dlnK_c^*}{dT} = \Delta H^* + RT \tag{3}$$

By combining equations (1), (2) and (3), the following equation is deduced:

$$A = \frac{k_B T}{h} \cdot e \cdot e^{\frac{\Delta S^*}{R}} \tag{4}$$

where k_B is Boltzmann constant, h is Planck constant, K_c^* is the equilibrium constant for reactions involving transition states, and R is gas constant. According to equations (1), (2) and (4), E_a and ΔS^* can be derived by fitting the Arrhenius relationship. Therefore, the effect of different quaternary ammonium cations on the reaction activity can be understood by investigating these parameters.

We measured R_{ct} values over CP and CP-CTAB at different temperatures, and attained a linear relationship between ln(1/R_{ct}) and 1/T (Supplementary Figs. 28, 29), in consistent with previous reports[10, 11]. By linking ln(1/R_{ct})-1/T linear relationship with Arrhenius equation (ln$k$-1/T), we tentatively associate 1/R_{ct} with k (rate constant), and k can be presented as the following equation (5):

$$k = Ke^{-\frac{\alpha F \varphi}{RT}} \propto \frac{1}{R_{ct}} \tag{5}$$

where K is the thermal rate constant without applied potential (φ = 0), F is Faradaic constant, α is transfer coefficient and φ is applied potential. The ψ_1 effect (i.e., Frumkin correction) can be ignored by ruling out the effect of local electric field induced by CTAB in the discussion of the manuscript. Therefore, we deduce that R_{ct} is influenced by the thermal rate constant (K) without applied potential (φ = 0), and K is related to E_a of benzaldehyde C–C coupling reaction without external potential.

Supplementary Note 6: the effect of electrolytes with different alkali cations

Li_2SO_4, K_2SO_4 and Cs_2SO_4 at the same concentration (0.5 M; due to solubility limitation, the actual concentration of Li_2SO_4 was about 0.25 M) were used, respectively, as substitutes for Na_2SO_4 electrolyte. Then, electrochemical pinacol coupling was conducted with or without CTAB. The catalytic results are shown in the revised Supplementary Fig. 36.

Without CTAB, the reaction rate followed the trend of $Li^+ < Na^+ < K^+ \approx Cs^+$. The dr of hydrobenzoin followed the trend of $Na^+ \approx K^+ < Li^+ < Cs^+$. These results indicate that different cations indeed affected the activity and dr, probably induced by adsorption at electrode interface. However, the increased extent of reaction rate was not as significant as that using CTAB (K^+ and Cs^+: 0.18 mmol h^{-1} cm^{-2}; CTAB: 0.28 mmol h^{-1} cm^{-2}), suggesting the important role of CTAB for the enhanced activity. Regarding dr value, the addition of Cs^+ promoted dr (2.14) even higher than the addition of CTAB (1.82), while other cations did not exhibit a significant effect. These results can be explained by the specific adsorption of Cs^+ at electrode interface to repel the interfacial water, as evidenced by the desorption peak at −0.8 V versus Ag/AgCl in the LSV curve (the revised Supplementary Fig. 36a, b). We tentatively proposed that Cs^+ may display a similar effect as CTA^+, that is, creating a hydrophobic microenvironment at electrode−electrolyte interface.

When CTAB was introduced into the electrolyte involving Li^+, Na^+, K^+ or Cs^+ cations, the reaction rate exhibited an order of $Li^+ < Na^+ \approx K^+ < Cs^+$ but with insignificant difference. The dr of hydrobenzoin was basically unchanged between different alkali cations. These results indicate that the specific adsorption of CTA^+ was more favorable compared with that of other cations we tested.

Collectively, the above experiments indicate CTAB serves as a unique molecule for microenvironment regulation during electrochemical pinacol coupling reaction. In addition, the varied dr induced by Cs^+ is worth further exploration, and its in-depth investigation is beyond the scope of this manuscript.”

2. Supplementary Figures

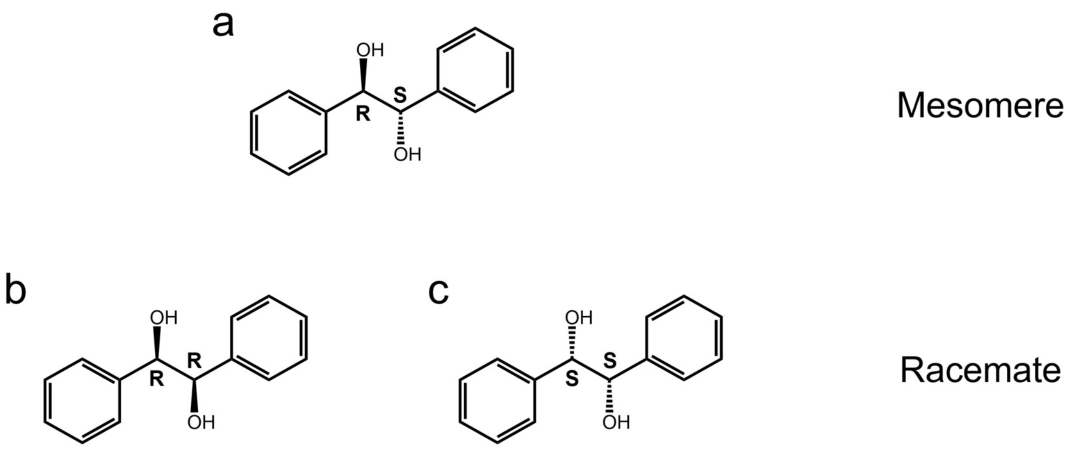

Supplementary Fig. 1 Chemical structure of stereoisomers of hydrobenzoin. Structure diagram of hydrobenzoins with different stereo-structures: mesomere (**a** RS) and racemate (**b** RR, **c** SS).

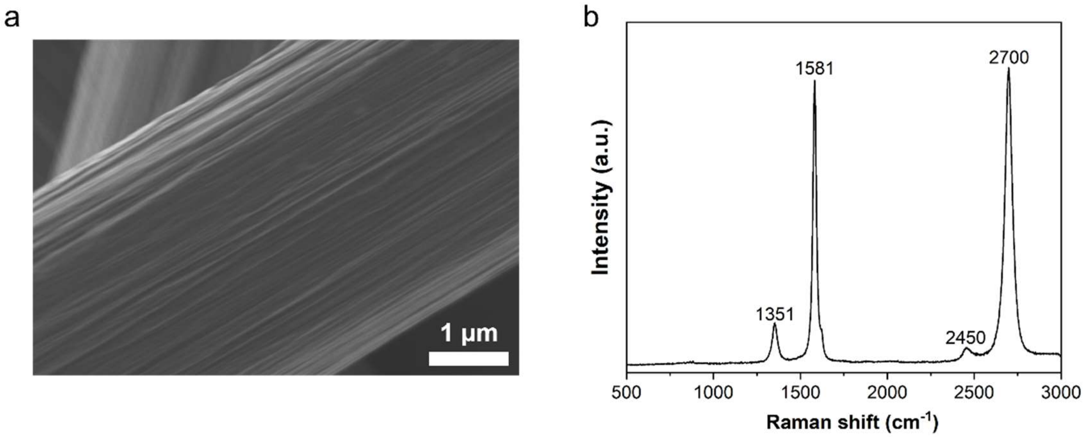

Supplementary Fig. 2 Characterization of acid-treated CP. a SEM image and **b** Raman spectra of acid-treated CP.

The Raman peaks at 1351, 1581, 2450 and 2700 cm^{-1} are assigned to the D-band, G-band, D’ band and 2D band of carbon[12, 13], respectively.

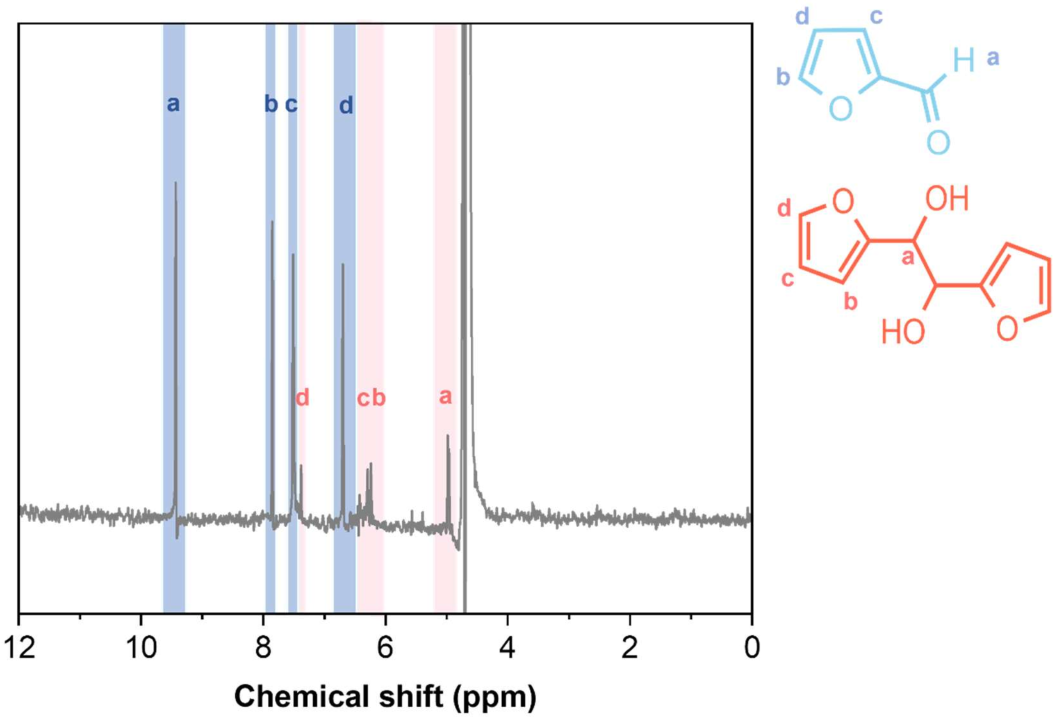

Supplementary Fig. 3 ^{1}H-NMR spectrum of the reaction after bulk electrolysis of furfural over CP electrode. The reaction was conducted at –1.4 V vs. Ag/AgCl. The obtained spectrum is consistent with that in the previous report[14]. The chemical structure of furfural and 1,2-di(2-furyl)-1,2-ethanediol are shown below.

Furfural: ^{1}H-NMR (10% D_2O) δ 6.69 (m, 1H), 7.50 (m, 1H), 7.85 (m, 1H), 9.43 (s, 1H). 1,2-Di(2-furyl)-1,2-ethanediol: ^{1}H-NMR (10% D_2O) δ 4.95 (s, 2H, racemate), 4.99 (s, 2H, mesomer), 6.23 (m, 2H), 6.31 (m, 2H), 7.37 (m, 2H).

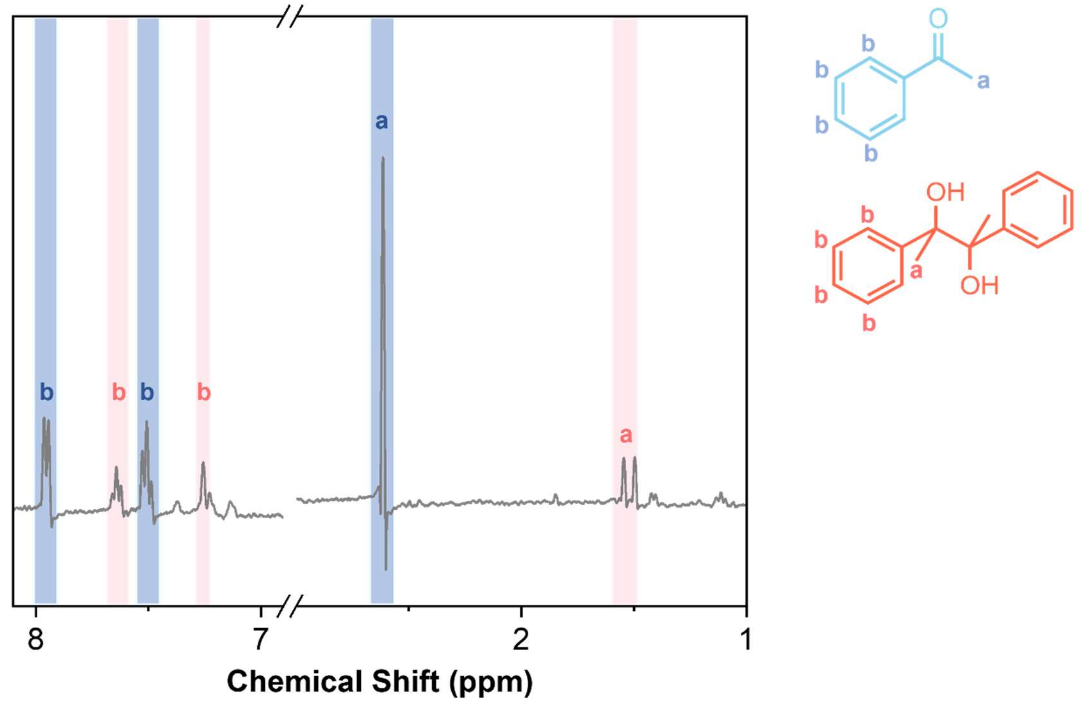

Supplementary Fig. 4 ^{1}H-NMR spectrum of the reaction after bulk electrolysis of acetophenone over CP electrode. The reaction was conducted at –1.6 V vs. Ag/AgCl. The chemical structure of acetophenone and 2,3-diphenyl-2,3-butanediol are shown below.

Acetophenone: ^{1}H-NMR (10% D_2O) δ 2.61 (s, 3H), 7.51-7.95 (m, 5H).
2,3-Diphenyl-2,3-butanediol: ^{1}H-NMR (10% D_2O) δ 1.49 (s, 6H, racemate), 1.55 (s, 6H, mesomer), 7.26-7.64 (m, 10H).

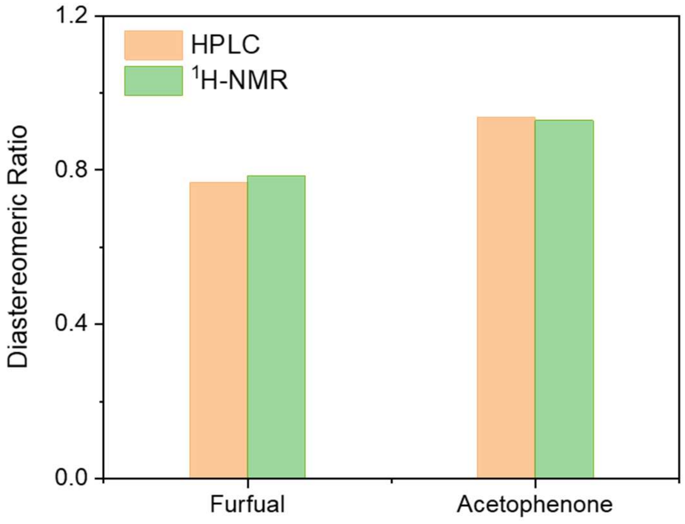

Supplementary Fig. 5 Comparison of dr value determined by HPLC and ^{1}H-NMR. Bulk electrolysis of furfural and acetophenone over CP-CTAB were performed, and the products were analyzed by HPLC and ^{1}H-NMR.

According to the report of Kim and coworker[15], the stereoisomers of the reduction coupling products of aromatic aldehydes and ketones can be qualitatively and quantitively identified by ^{1}H-NMR. It is demonstrated that the chemical shift of directly-bonded hydrogen atoms (for aldehydes) or hydrogen atoms on methyl group (for ketones) connected to the chiral center on the mesomers is 0.05~0.10 ppm higher than that of racemate.

Hence, we measured the dr value of coupling products of furfural and acetophenone by ^{1}H-NMR based on the qualitative method shown above. The results show a good consistent with the results measured by HPLC. Therefore, it is safe to qualitatively identify the stereoisomers of the coupling products from furfural and acetophenone by ^{1}H-NMR, and to quantitatively measure the reaction results by HPLC

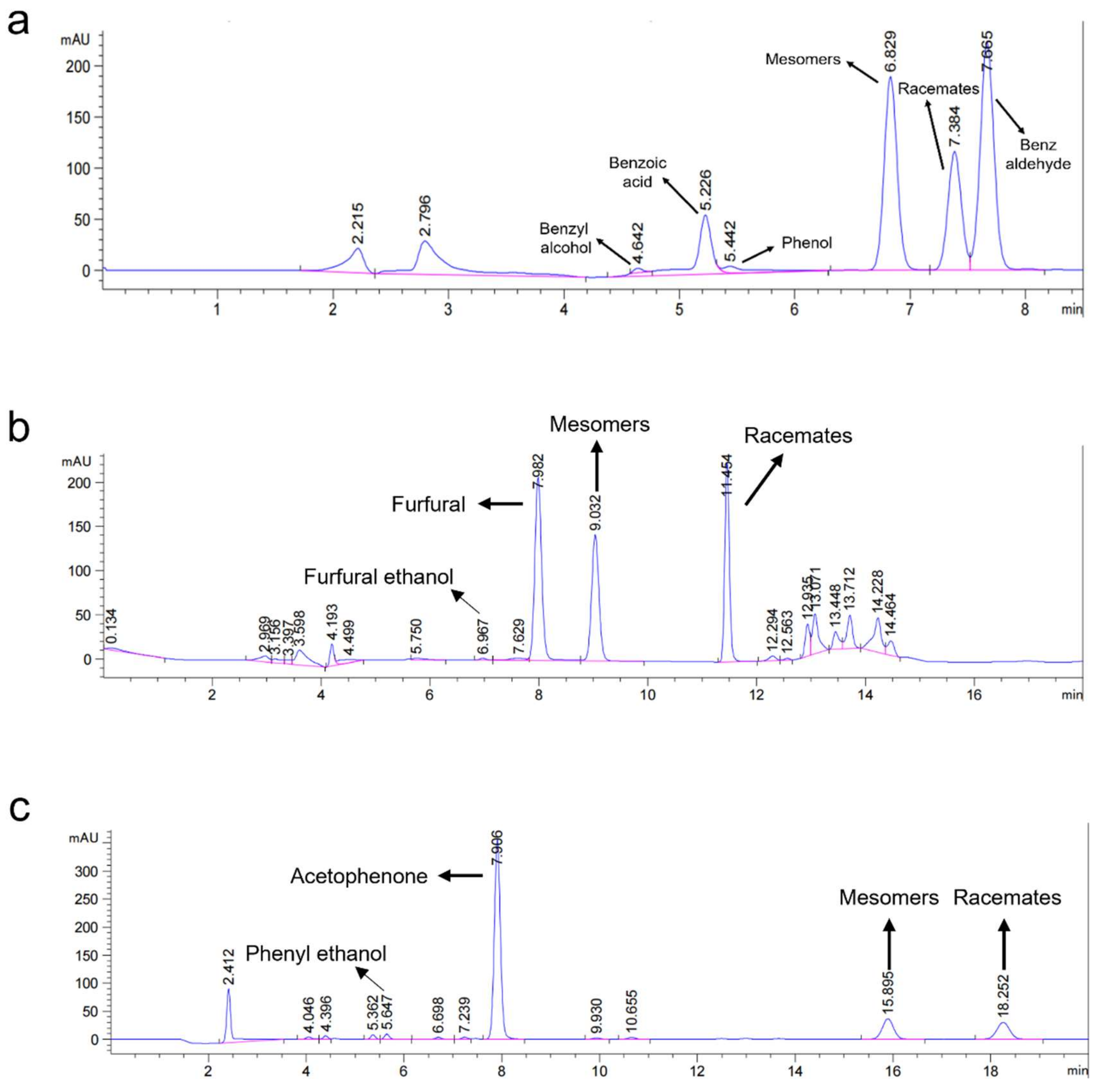

Supplementary Fig. 6 HPLC spectra of the reaction products. HPLC spectra of the reaction products with **a** benzaldehyde, **b** furfural and **c** acetophenone. The reactions were carried out in 0.5 M Na_2SO_4 electrolyte containing 25 mM substate.

.

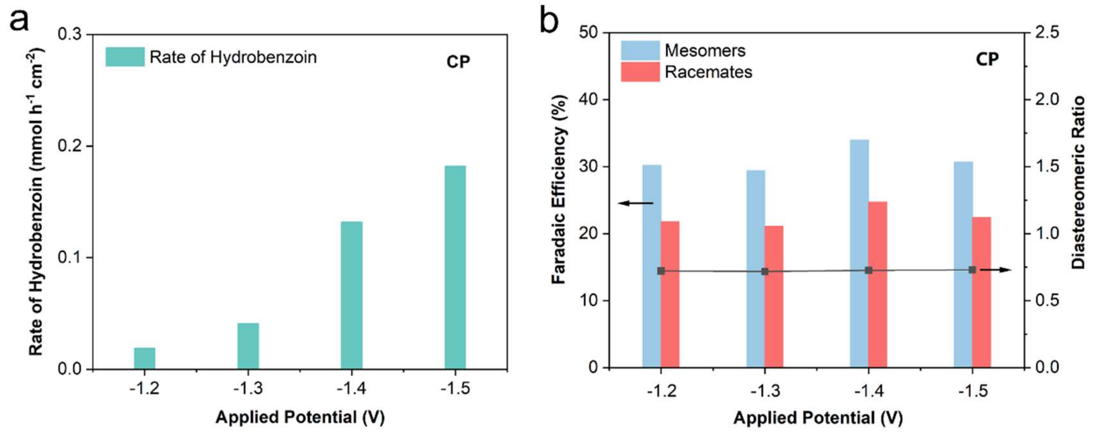

Supplementary Fig. 7 Catalytic performances of CP at different potentials. a Reaction rate and **b** stereoselectivity of hydrobenzoin over CP electrode at different potentials.

The stereoselectivity of coupling products hardly changes with the applied potentials. This indicates that the stereoselectivity is not directly affected by the potential, and the stereoselectivity of CP-CTAB system varying with the potential come from the interface behaviors of CTAB changing with the potential.

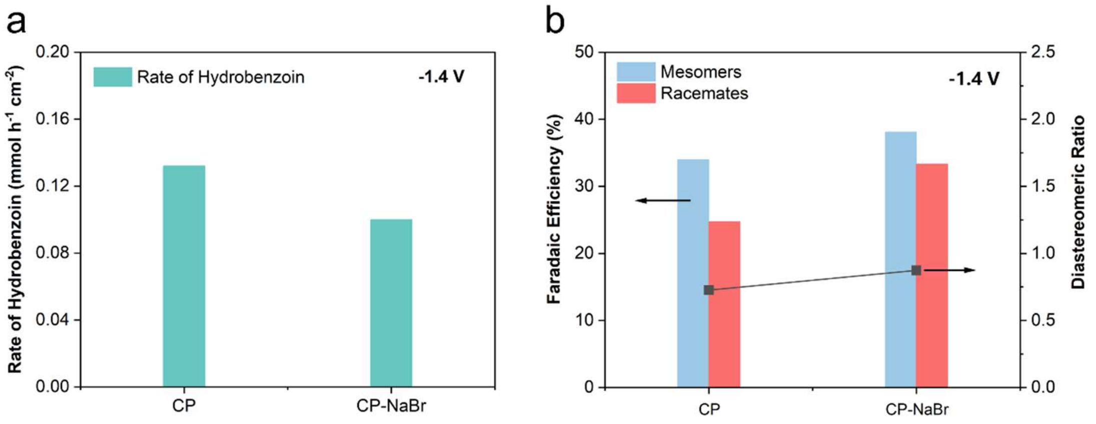

Supplementary Fig. 8 Catalytic performances of CP and CP-NaBr electrodes. a Reaction rate and **b** stereoselectivity of hydrobenzoin over CP and CP-NaBr electrode at −1.4 V versus Ag/AgCl.

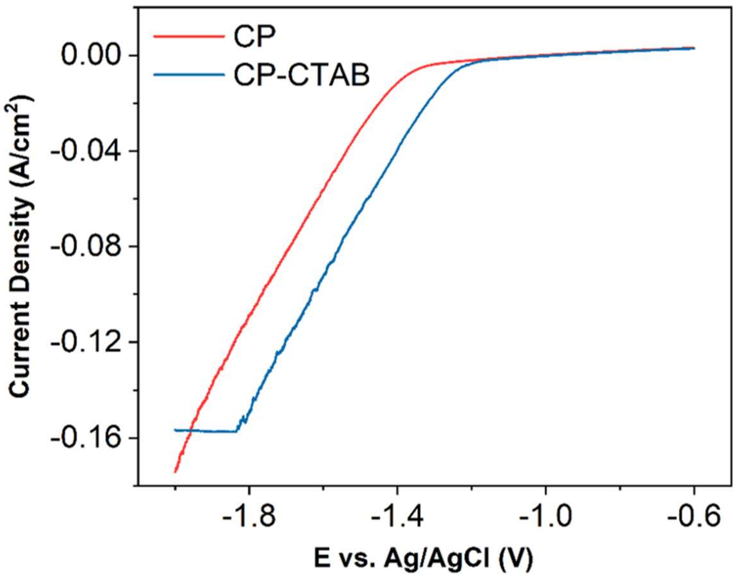

Supplementary Fig. 9 Linear sweep voltammetry (LSV) curves. LSV curves in 0.5 M Na_2SO_4 and 50 mM benzaldehyde electrolyte with and without 1 mM CTAB from −2.0 to −0.6 V versus Ag/AgCl at the scan rate of 50 mV s^{-1}.

To reveal the promoting effect of CTAB, we first investigated whether CTAB is adsorbed at CP surface. The LSV curves show that CP-CTAB displays higher polarization current compared to CP, implying the adsorption of CTAB in the potential range.

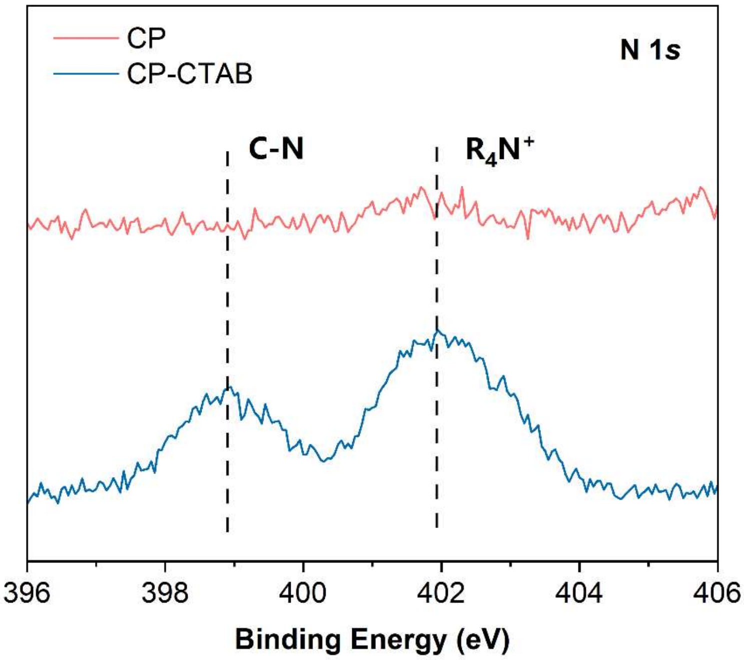

Supplementary Fig. 10 X-ray photoelectron spectroscopy (XPS) spectra. N 1*s* XPS spectra of CP and CP-CTAB electrodes.

XPS spectra of post CP-CTAB (the electrode after catalytic reaction) shows a peak at 401.9 eV in N 1*s* spectra, which is attributed to the CTA^+ head group of CTAB,[16] and a peak at 398.9 eV that is assigned to the cation-π interaction between the head group of CTAB and CP.[17]

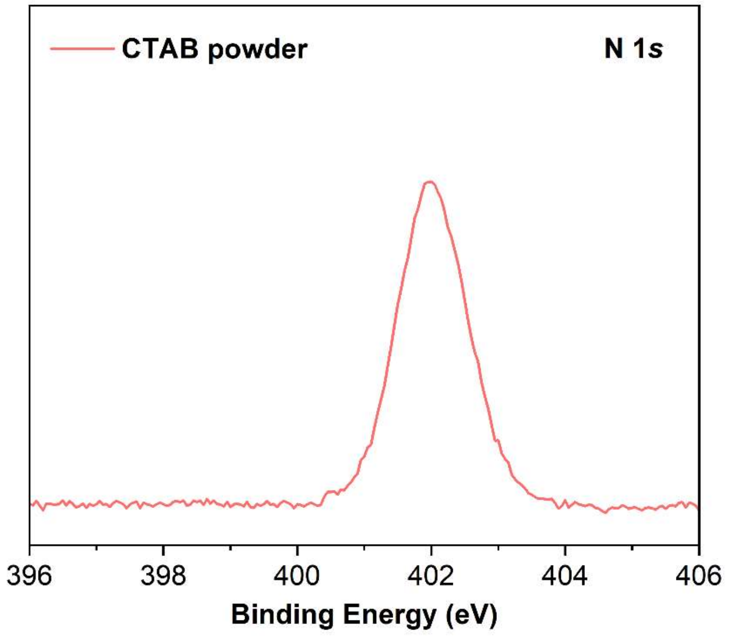

Supplementary Fig. 11 X-ray photoelectron spectroscopy (XPS) spectra. N 1*s* XPS spectra of CTAB powder. The peak at 402.0 eV is assigned to the CTA^+ group of CTAB.

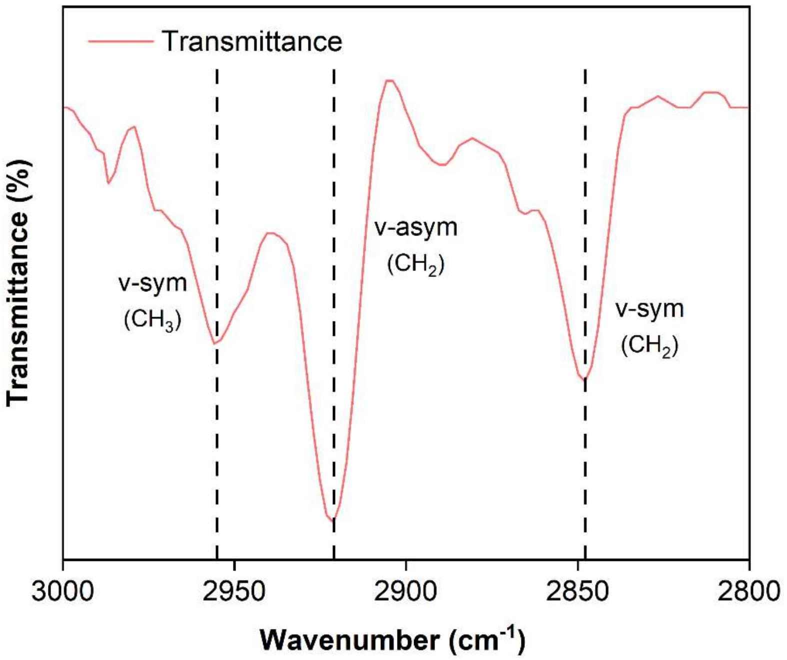

Supplementary Fig. 12 Infrared spectroscopy (IR) spectra. Infrared spectra of CP-CTAB system at –1.4 V versus Ag/AgCl in the range of 3000~2800 cm^{-1}.

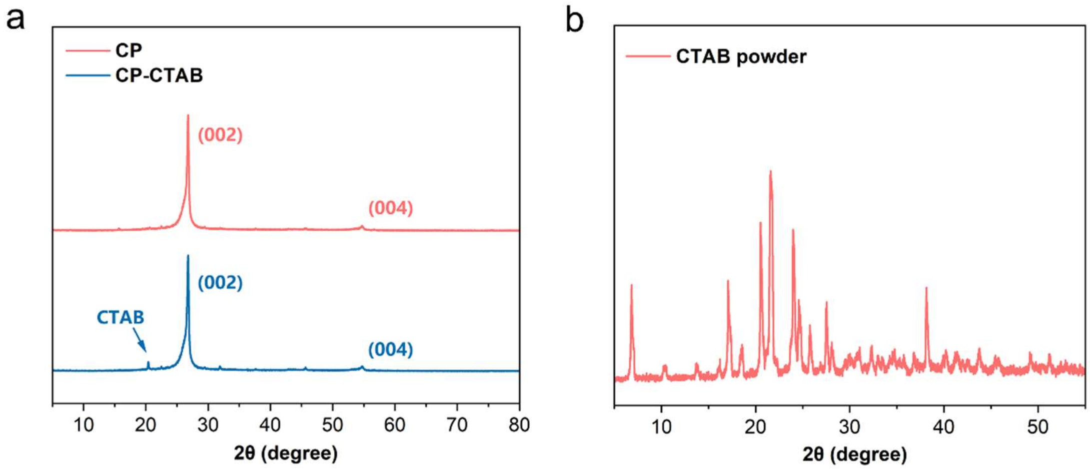

Supplementary Fig. 13 X-ray diffraction (XRD) spectra. XRD patterns of **a** CP and CP-CTAB electrodes as well as **b** CTAB powder.

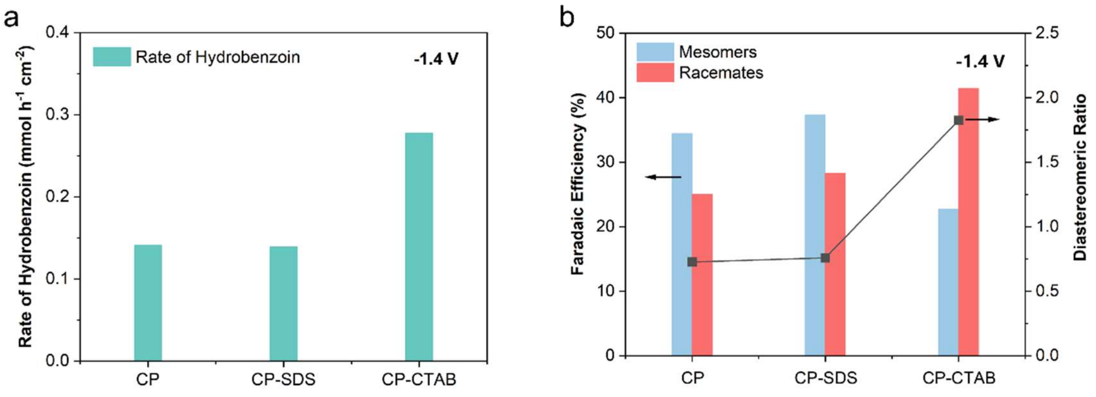

Supplementary Fig. 14 Catalytic performances of CP, CP-SDS, CP-CTAB electrodes. a Reaction rate and **b** stereoselectivity of hydrobenzoin over CP, CP-SDS, CP-CTAB electrodes at −1.4 V versus Ag/AgCl.

It can be observed that SDS with negatively charged head group has little influence on the reaction activity and stereoselectivity, indicating that the specific adsorption of surfactants resulting from the coulomb factor is necessary for the enhancement of reaction activity and stereoselectivity.

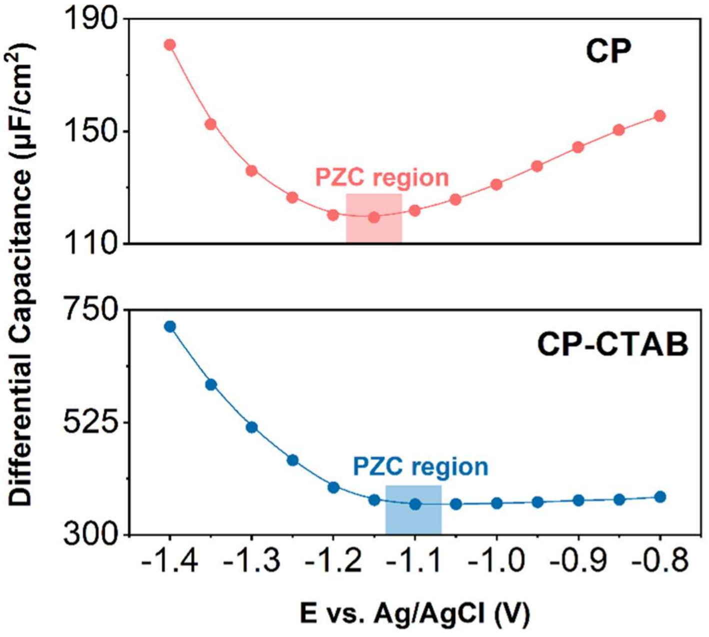

Supplementary Fig. 15 Differential capacitance results. Differential capacitance plots over CP and CP-CTAB electrodes in 0.1 M Na_2SO_4 electrolyte.

Differential capacitance (C_{diff}) curve was measured because it can reveal the structures of electric double layer at different potentials. We used relatively low concentration electrolyte (0.1M Na_2SO_4), since the point of zero charge (PZC) derived from differential capacitance plot is difficult to identify at high concentration. For bare CP, the PZC locates at −1.15 V, indicating that CP interface is negatively charged under electrochemical condition (−1.4 V versus Ag/AgCl), enabling the adsorption of CTAB with its positively-charged head group[18]. We then assessed PZC of CP-CTAB, showing positive shift compared with that of CP (from −1.15 to −1.1 V). Considering that a more positive potential is required to achieve zero charge when more charge is accumulated at the interface, the PZC results suggest that CTAB presents specific adsorption over CP via its positively-charged head group.

The enhancement of adsorption strength of CTAB is further demonstrated by the larger surface charge density (|σ|, see Supplementary Note 1 for calculation details). The |σ| at a certain potential (E_1) is calculated by integrating the differential capacitance (C_{diff}) from the E_{PZC} to E_1 according to equation (6).

$$|\sigma| = | \int_{E_{\mathrm{PZC}}}^{E_1} \mathrm{C_{diff}}\, \mathrm{d}E \,| \tag{6}$$

The obtained larger |σ| for CP-CTAB compared with that for CP indicates that the charge density over CP interface notably increases when CTAB is present, corroborating that CTAB is adsorbed on CP with its positively-charged head group under reaction conditions.

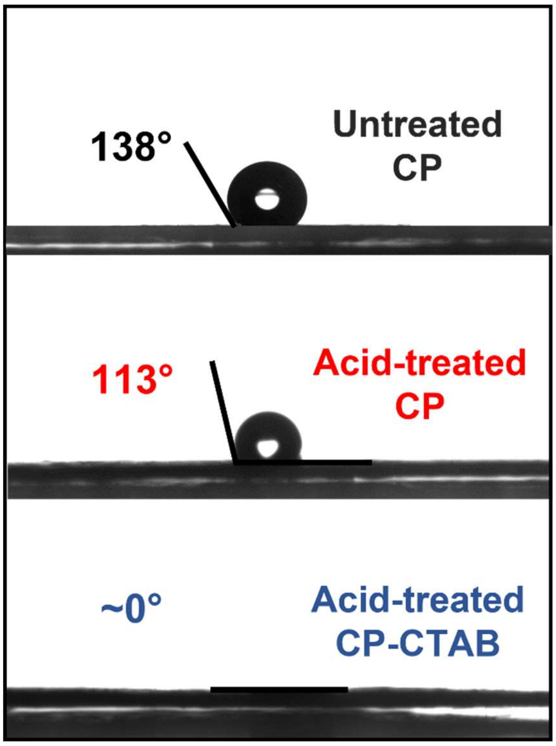

Supplementary Fig. 16 Contact angle experiments. Contact angle experiments of untreated CP, acid-treated CP and acid-treated CP-CTAB electrodes (from top to bottom). The photographs were captured at the moment when the water drop contacted with the carbon paper.

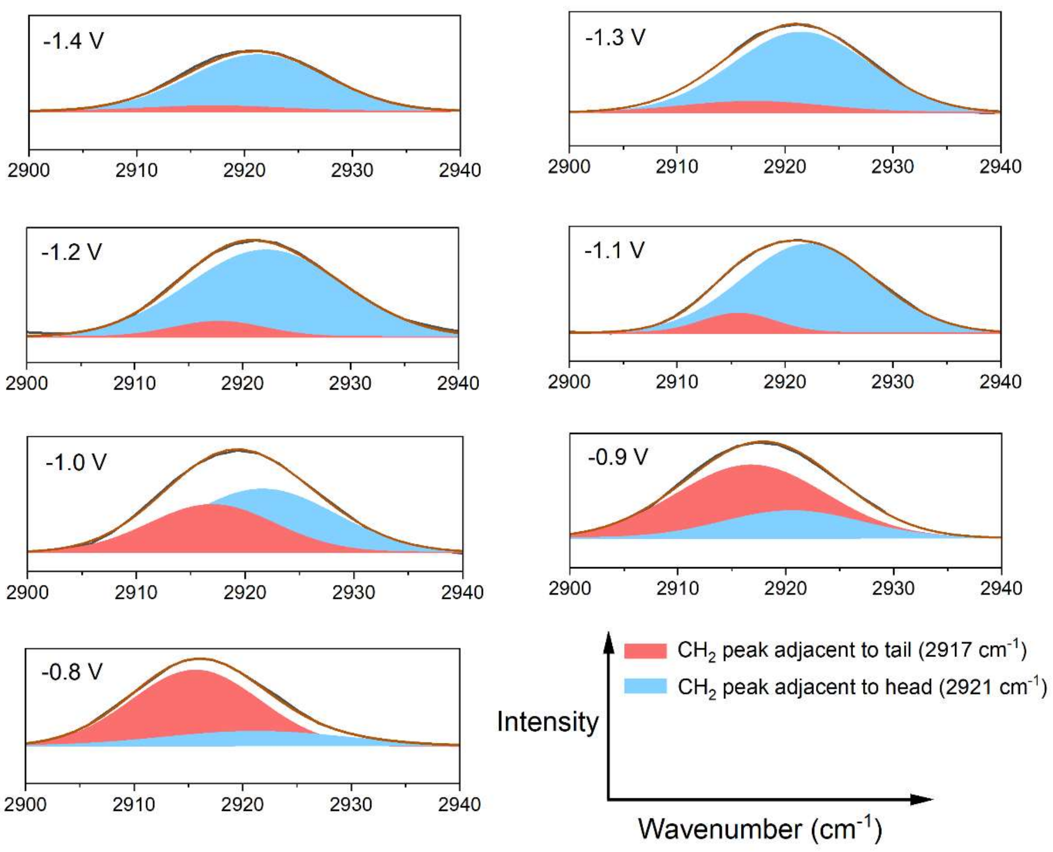

Supplementary Fig. 17 Deconvolution results of infrared peaks at different potentials. In-situ ATR SEIRAS spectra of asymmetric C−H stretching vibrations of CH_2 species at CP-CTAB electrode. The complete spectra are shown in Fig. 3a.

The C−H stretching band of CH_2 species (taking asymmetric C-H stretching band as examples) can be deconvolved into two distinct components involving 2916 and 2921 cm^{-1}, corresponding to the CH_2 species adjacent to the tail and head groups, respectively.

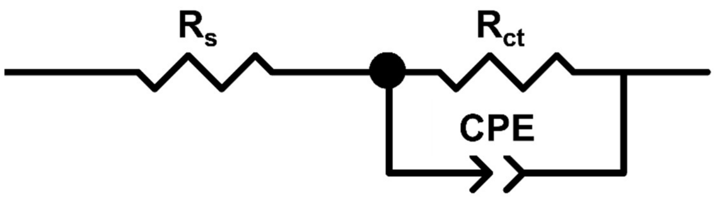

Supplementary Fig. 18 Equivalent circuit diagram. The equivalent circuit of all the systems.

The experimental results were analyzed by ZView 2 software using the above circuit, where R_s is the resistance of solution and R_{ct} is the resistance of charge transfer. CPE is the constant phase element.

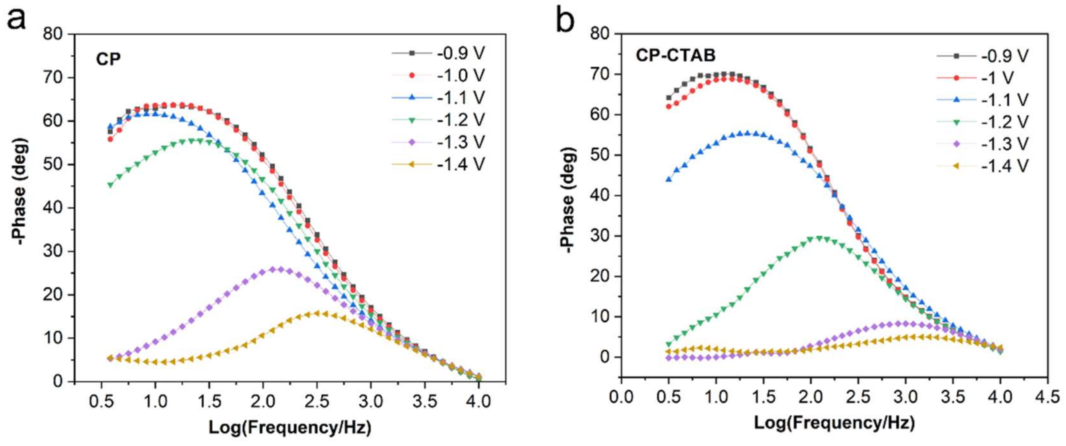

Supplementary Fig. 19 Electrochemical impedance spectroscopy (EIS) results with or without CTAB. Bode plots of **a** CP and **b** CP-CTAB systems at different potentials.

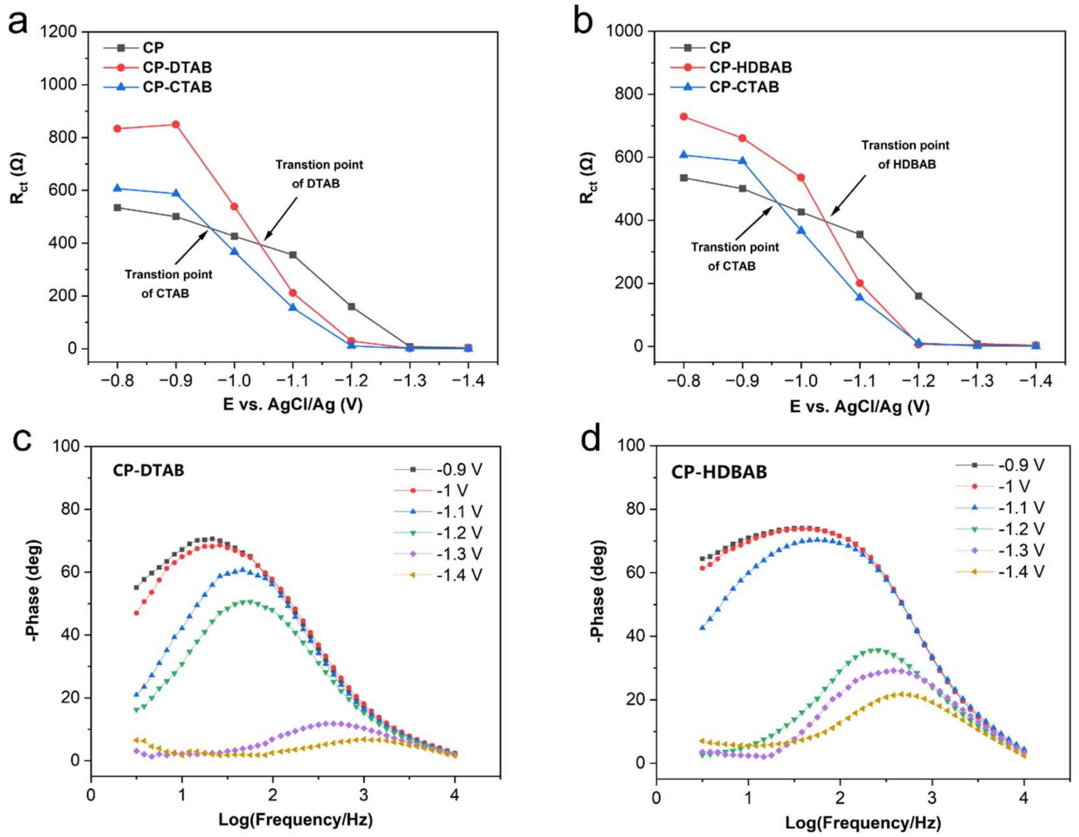

Supplementary Fig. 20 Electrochemical impedance spectroscopy (EIS) results with CTAB, HDBAB and DTAB. a Resistance of charge transfer over CP, CP-DTAB and CP-CTAB electrodes at different potentials. **b** Resistance of charge transfer over CP, CP-HDBAB and CP-CTAB electrodes at different potentials. Bode plots of **c** CP-DTAB and **d** CP-HDBAB systems at different potentials.

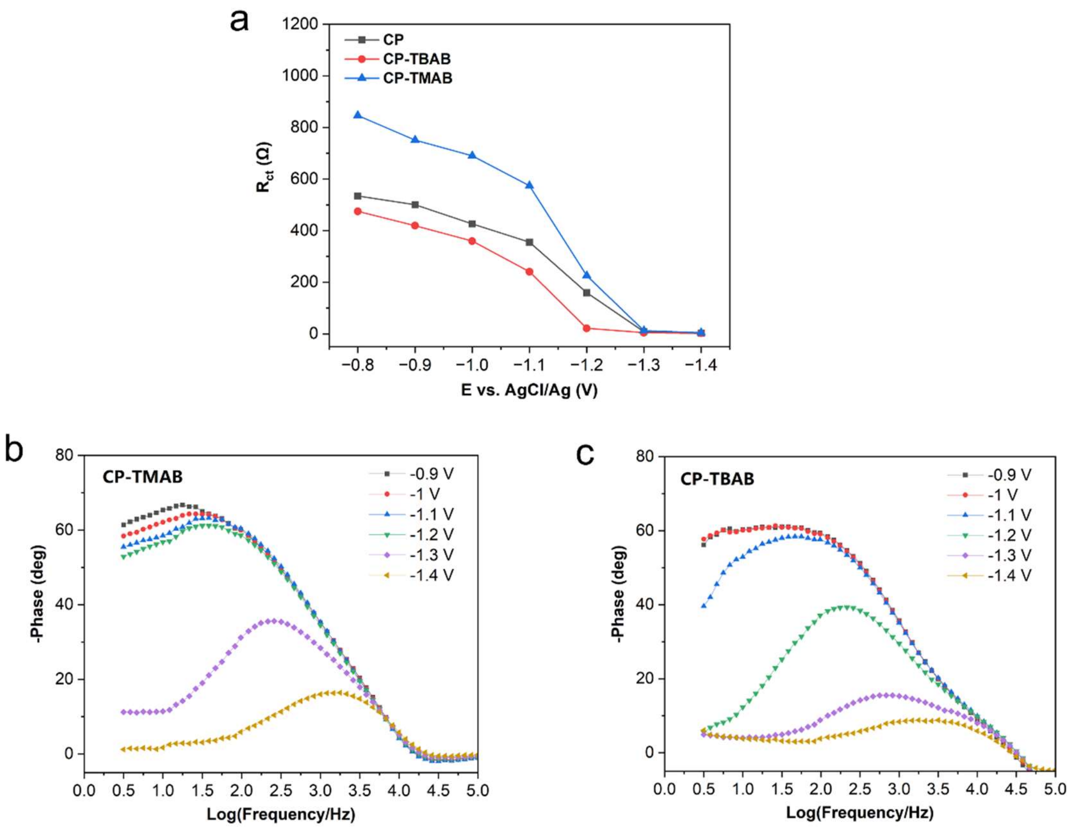

Supplementary Fig. 21 Electrochemical impedance spectroscopy (EIS) results with TMAB and TBAB. a Resistance of charge transfer over CP, CP-TMAB and CP-TBAB electrodes at different potentials. Bode plots of **b** CP-TMAB and **c** CP-TBAB systems at different potentials.

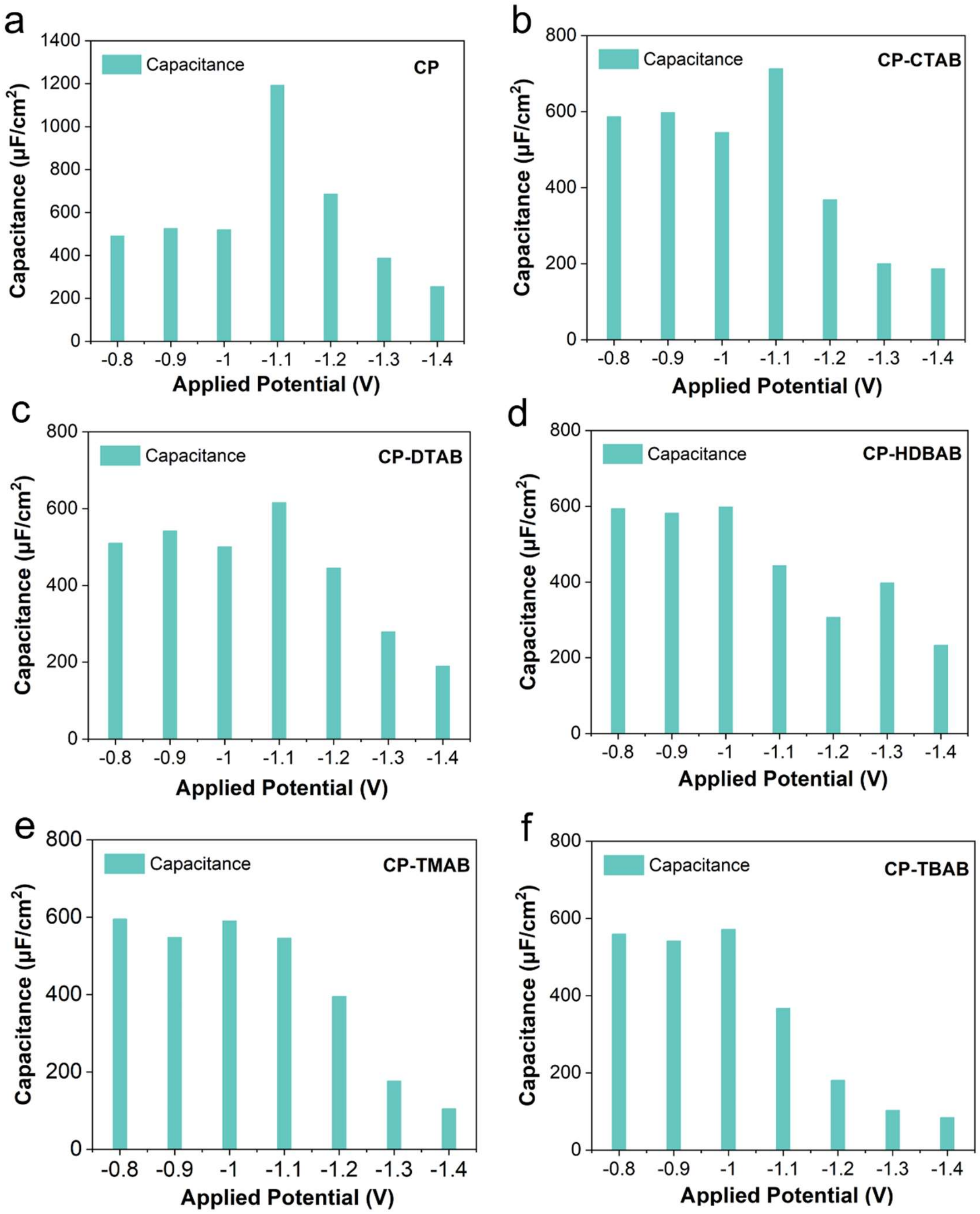

Supplementary Fig. 22 Capacitance results from electrochemical impedance spectroscopy (EIS) experiments. Capacitance obtained from the EIS data (Supplementary Tables 2-7) fitting by Zview 2 with **a** CP, **b** CP-CTAB, **c** CP-DTAB, **d** CP-HDBAB, **e** CP-TMAB, **f** CP-TBAB electrodes.

It can be observed that the capacitance values measured in the range of –0.8 to –1.0 V versus Ag/AgCl are maintained at about 500 to 600 μF/cm^2 for all the systems. It indicates that all the quaternary ammonium cations were desorbed within the above potential range, thus showing similar interface charge distribution to CP electrode without any modification.

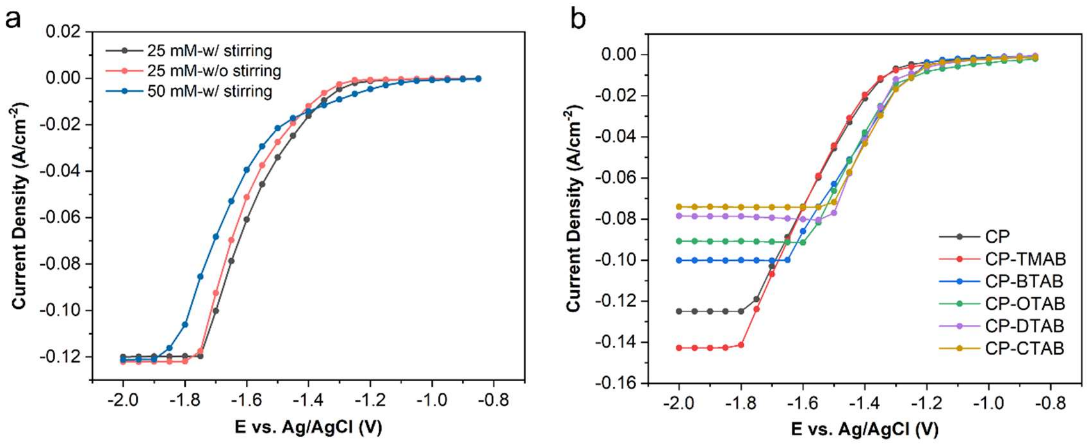

Supplementary Fig. 23 Normal pulse voltammetry (NPV) under different conditions. a NPV plots over CP-CTAB with different concentration of benzaldehyde (25, 50 mM) with or without stirring. Specifically, the reactions were carried out under the conditions of 25 mM benzaldehyde with stirring speed of 800 rpm (black line), 25 mM benzaldehyde without stirring (red line), 50 mM benzaldehyde with stirring speed of 800 rpm (blue line). **b** NPV plots over CP modified with different quaternary ammonium cations.

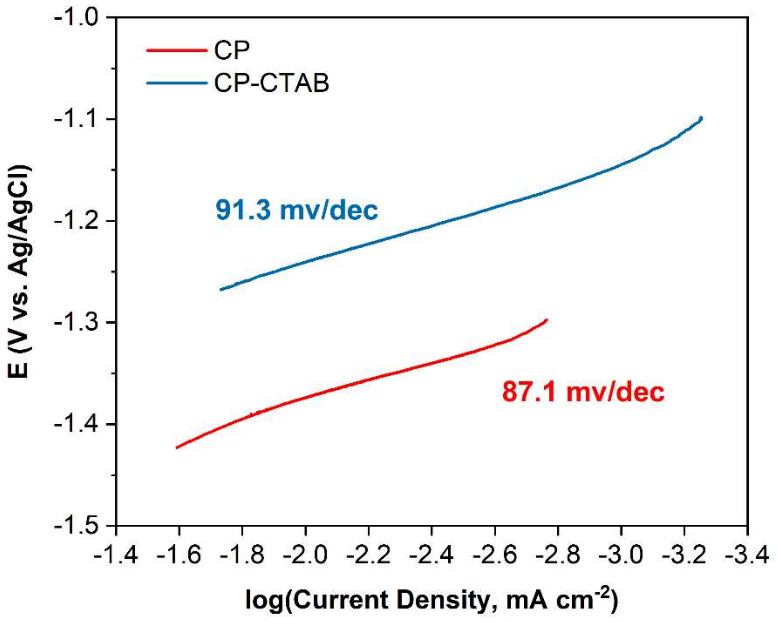

Supplementary Fig. 24 Tafel plots with i-R correction. Tafel plots with i-R correction of CP and CP-CTAB systems at a scan rate of 50 mV/s.

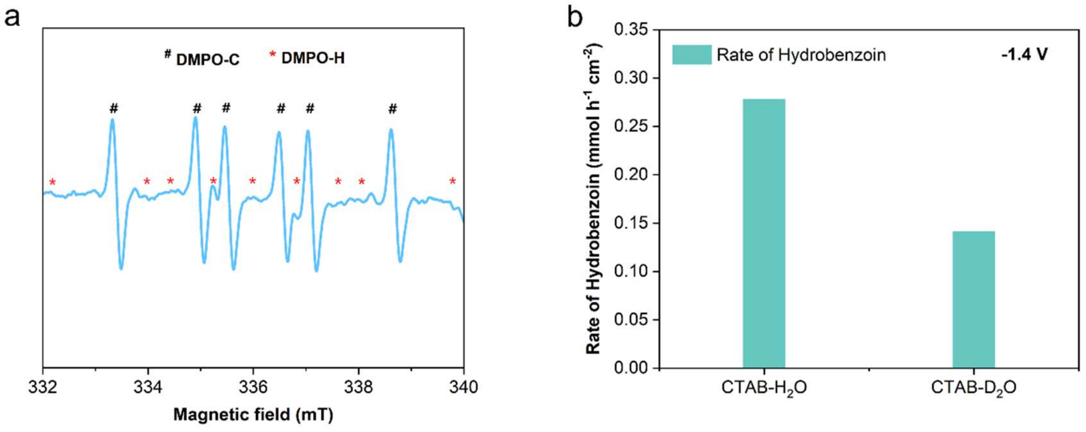

Supplementary Fig. 25 Mechanism studies. a Quasi-in situ electron paramagnetic resonance (EPR) spectra of CP-CTAB systems. **b** kinetic isotope effects (KIE) of CP-CTAB systems ($k_H/k_D = 1.99$)

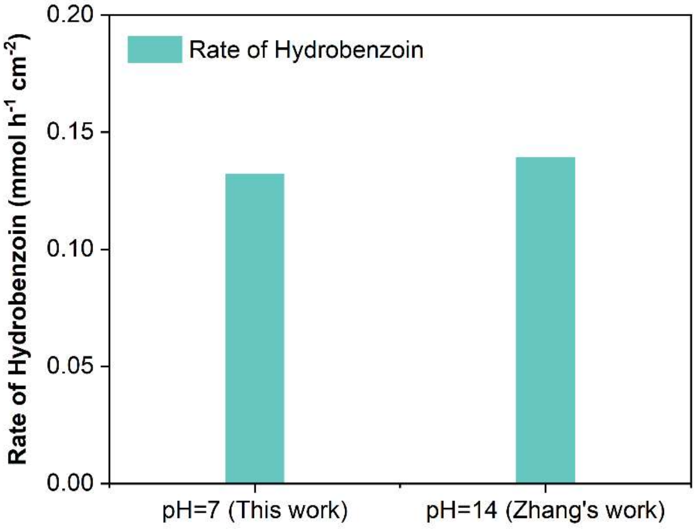

Supplementary Fig. 26 Catalytic comparison. Comparison of reaction rate between Zhang's work and this work using CP electrode[19].

For this reaction in neutral and alkaline media(pH>7), the RDS is demonstrated as the PCET process. On this occasion, water (rather than H_3O^+) participates in the reaction as the proton source. Therefore, the concentration of H_3O^+ is not included in the kinetic equation[20]. Hence, it is more reasonable to ignore the correction of potential by H^+ concentration and apply SHE scale rather than RHE scale for the comparison of reaction rate in neutral or alkaline media[21, 22].

In this work, the electrolysis experiments were performed in 0.5 M Na_2SO_4 electrolytes at −1.4 V versus Ag/AgCl, which can be converted to −1.203 V versus SHE. According to Zhang's work[19], we repeated the experiments in 1.0 M KOH electrolytes at −1.3 V versus Hg/HgO, which can be converted to −1.200 V versus SHE. The catalytic results show that the difference in reaction rate at the same potential versus SHE between the neutral (0.13 mmol h^{-1} cm^{-2}) and alkaline media (0.14 mmol h^{-1} cm^{-2}) is negligible, thus the influence of local pH can be ruled out.

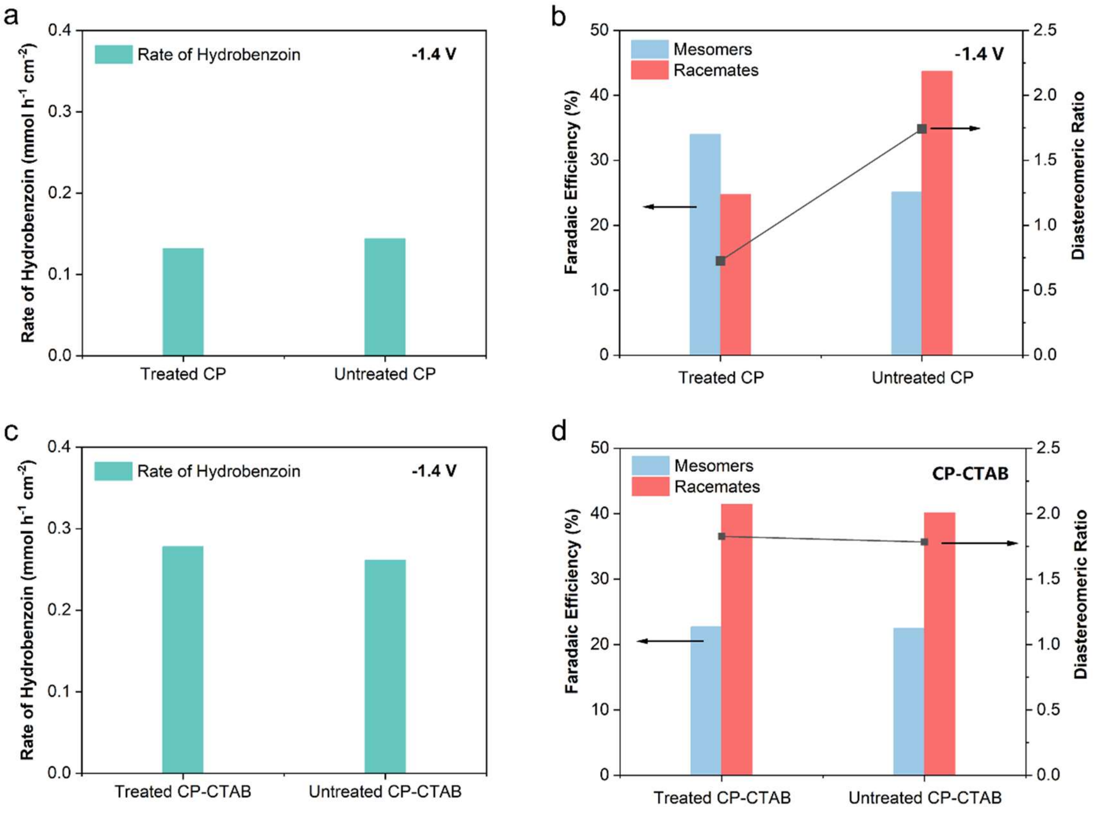

Supplementary Fig. 27 Catalytic performances of CP, CP-CTAB, acid-untreated CP and acid-untreated over CP-CTAB electrodes. a, c Reaction rate and **b, d** stereoselectivity of hydrobenzoin over CP and CP-CTAB electrodes with and without acid treatment at −1.4 V versus Ag/AgCl.

Compared with the acid-treated CP electrode with hydrophilic interface (shown in the above studies), we directly used acid-untreated CP that displays hydrophobic interface. The results show no obvious reaction rate difference between the acid-treated and acid-untreated CP, and also exhibit similar promoting degree before or after CTAB is introduced. These results demonstrate that the effect of hydrophobic interaction between the CP and ketyl radical may not be a decisive factor for activity enhancement.

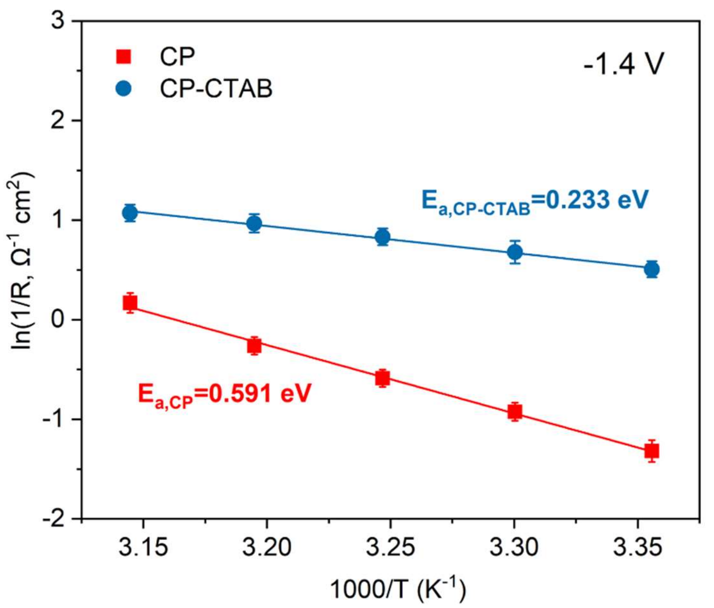

Supplementary Fig. 28 Arrhenius fitting plots of CP, CP-CTAB. Arrhenius plots for CP and CP-CTAB electrodes at −1.4 V versus Ag/AgCl calculated from the EIS curves. Error bars correspond to the standard deviation of three independent measurements.

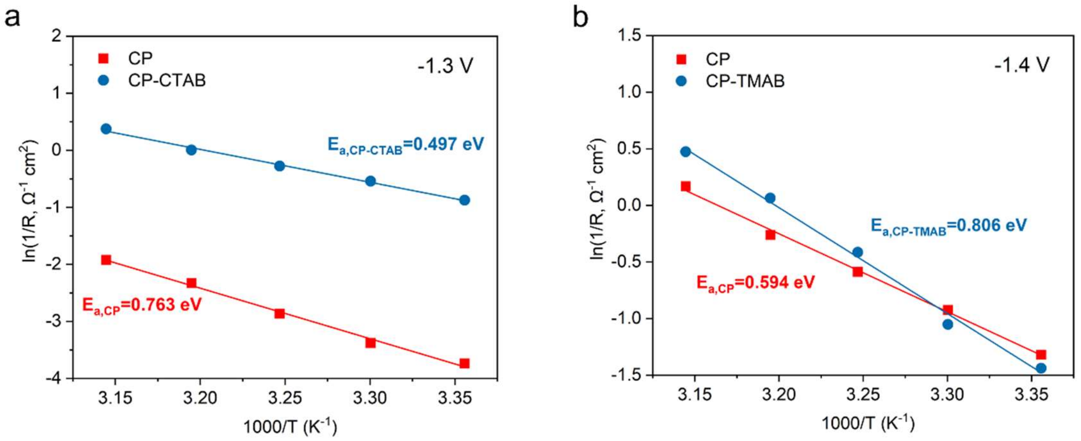

Supplementary Fig. 29 Arrhenius fitting plots of CP, CP-CTAB and CP-TMAB. Arrhenius plots for **a** CP and CP-CTAB electrodes at −1.3 V versus Ag/AgCl as well as **b** CP and CP-TMAB electrodes at −1.4 V versus Ag/AgCl calculated from the EIS curves.

For both CP and CP-CTAB electrodes, the activation energy values increase obviously at more positive potential (comparing −1.3 and −1.4 V versus Ag/AgCl,). Moreover, introduction of TMAB significantly increases the activation energy, which is assigned to the increased R_{ct} of CP-TMAB at room temperature.

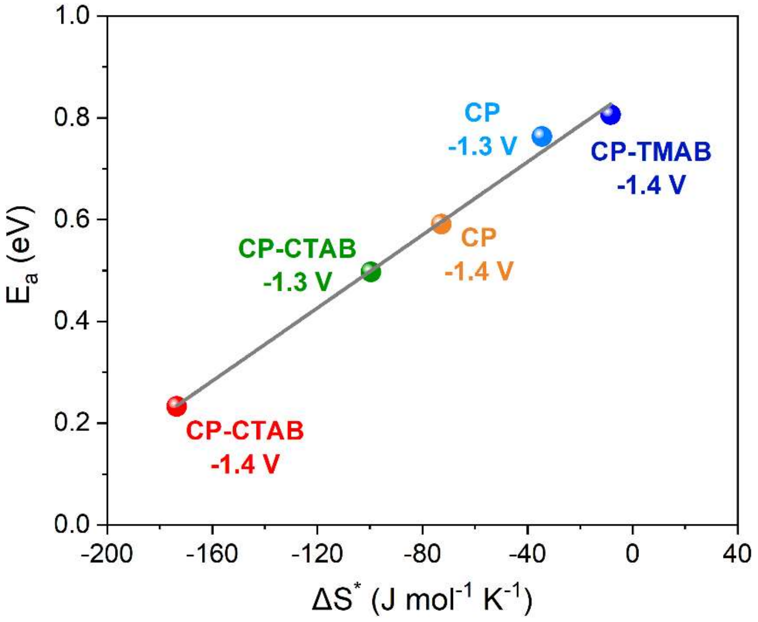

Supplementary Fig. 30 Enthalpy–entropy compensation plot. Relationship between activation energy (E_a) and activation entropy (ΔS^*) over different electrodes, which indicates a enthalpy–entropy compensation mechanism[23]. The tested potentials are shown in the figure.

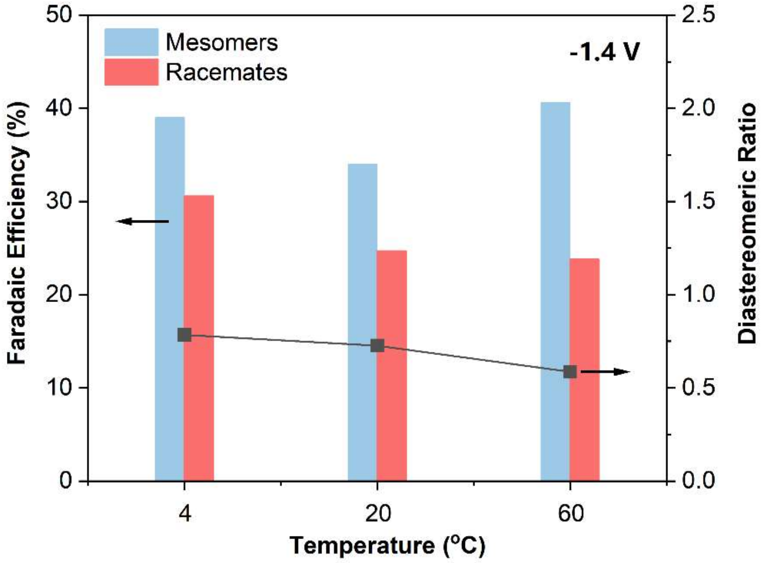

Supplementary Fig. 31 Stereoselectivity results of CP at different temperatures. Temperature effect on the stereoselectivity of hydrobenzoin over CP electrode at −1.4 V versus Ag/AgCl.

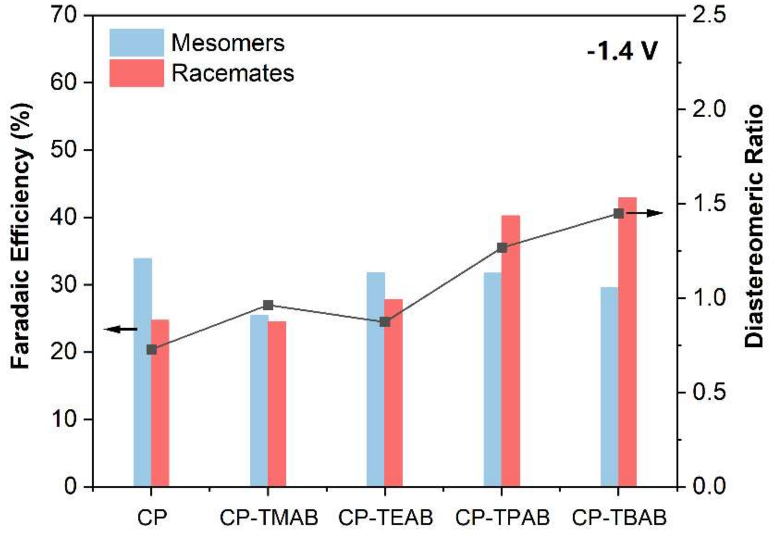

Supplementary Fig. 32 Stereoselectivity results of CP electrode modified with different spherical quaternary ammonium salts. Stereoselectivity of hydrobenzoin over CP electrode modified with different spherical quaternary ammonium salts at −1.4 V versus Ag/AgCl. The quaternary ammonium salts include tetramethyl ammonium bromide (TMAB), tetraethyl ammonium bromide (TEAB), tetra-n-propyl ammonium bromide (TPAB) and tetra-n-butyl ammonium bromide (TBAB).

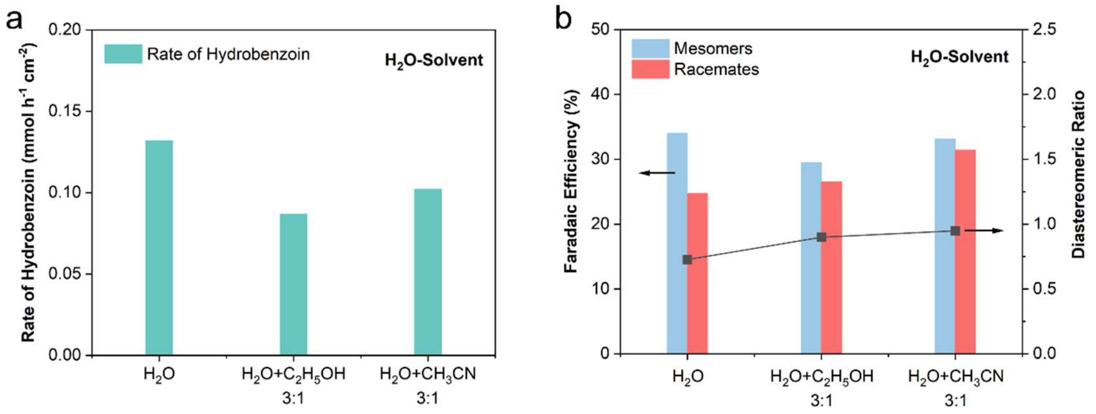

Supplementary Fig. 33 Catalytic performances of CP in different mixed solutions. **a** Reaction rate and **b** stereoselectivity of hydrobenzoin over CP in different mixed solvents over CP electrode at −1.4 V versus Ag/AgCl. The results showed that when the solvation ability of the mixed solvent decreases, the stereoselectivity of racemate gets improved.

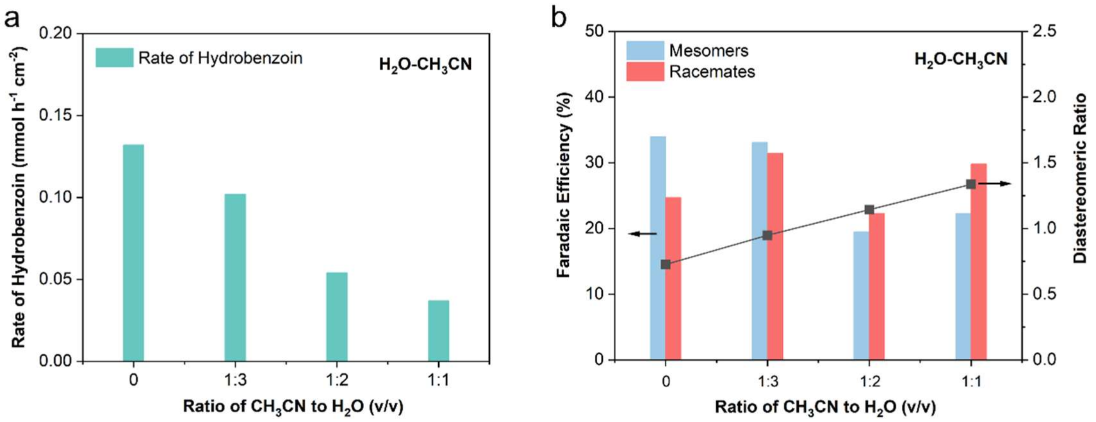

Supplementary Fig. 34 Catalytic performances of CP in CH_3CN-H_2O mixed solution with different ratios (v/v). a Reaction rate and **b** stereoselectivity of hydrobenzoin over CP in CH_3CN-H_2O mixed solution with different CH_3CN/H_2O ratios (v/v) over CP electrode at −1.4 V versus Ag/AgCl. When the volume fraction of CH_3CN increased, the stereoselectivity of racemate was improved.

When the ratio of CH_3CN to H_2O is higher than 1:2, Na_2SO_4 in the solution will be spontaneously extracted, which leads to the reduction of electrolyte concentration. To eliminate its effect on stereoselectivity, we carried out bulk electrolysis in the Na_2SO_4 solution with different concentrations.

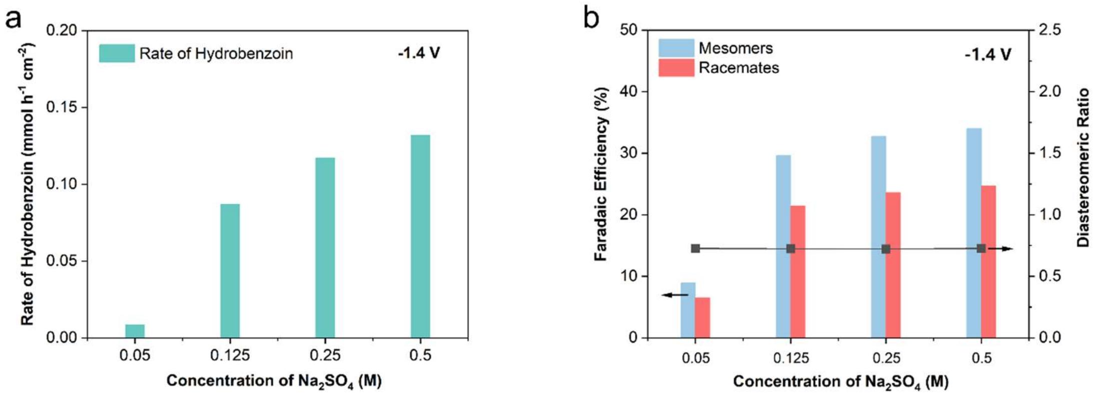

Supplementary Fig. 35 Catalytic performances of CP in Na_2SO_4 with different concentrations. a Reaction rate and **b** stereoselectivity of hydrobenzoin over CP electrode at −1.4 V versus Ag/AgCl in Na_2SO_4 electrolytes with different concentrations.

The results indicate that the concentration of Na_2SO_4 will not influence the stereoselectivity of hydrobenzoin. Together with the results of stereoselectivity at different potentials over CP electrode (Supplementary Fig. 7), it demonstrates that the change of electric double layer (EDL) does not significantly influence the stereoselectivity of hydrobenzoin without CTAB.

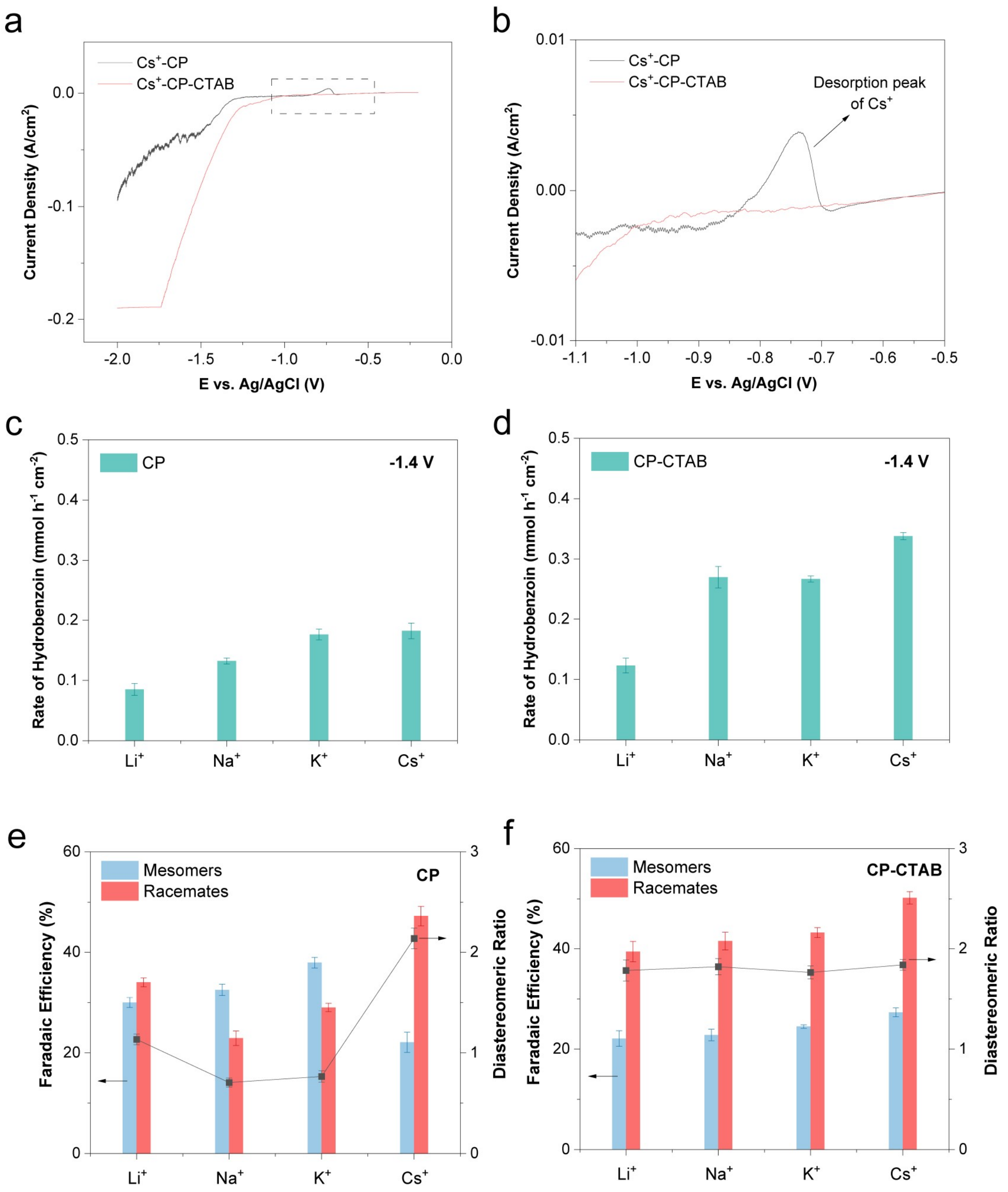

Supplementary Fig. 36 Investigation of different alkali cations on affecting electrochemical pinacol C−C coupling reaction. **a** LSV plot and **b** corresponding enlarged region. Reaction conditions: electrolyte contains 0.5 M Cs_2SO_4 and 25 mM benzaldehyde, with or without CTAB. Reaction rate of hydrobenzoin in the electrolyte with 0.5 M different alkali cations (Li^+, Na^+, K^+ or Cs^+) at −1.4 V versus Ag/AgCl **c** without or **d** with 1 mM CTAB. Stereoselectivity of hydrobenzoin in the electrolyte with different cations at −1.4 V versus Ag/AgCl **e** without or **f** with 1 mM CTAB. Error bars correspond to the standard deviation of three independent measurements.

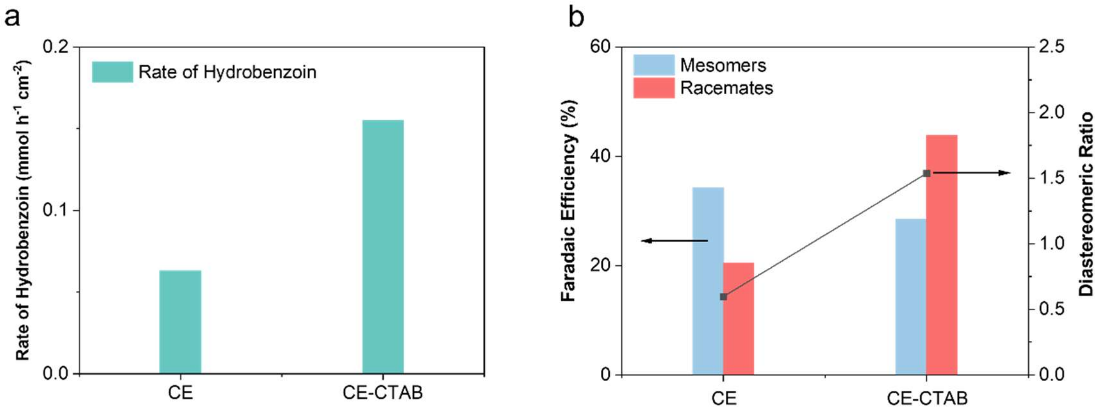

Supplementary Fig. 37 Catalytic performance of Carbon ECP600JD (CE) with or without CTAB. a Reaction rate and **b** stereoselectivity of hydrobenzoin over CE and CE-CTAB electrodes at –1.4 V versus Ag/AgCl. In the ATR-SERIRS experiments, Ketjen black powder (Carbon ECP600JD, abbreviated as CE) was used as a substitute for carbon paper.

The results show that a promoted activity and varied dr value are observed over CE electrode, similar with that over CP electrode (Figs. 2a, b), demonstrating that the conclusion of ATR-SERIRS over CE electrode is valid to explain the catalytic results over CP electrode.

3. Supplementary Tables

Supplementary Table 1. Simulated impedance parameters of Bode plots at –1.4 V versus Ag/AgCl.

Systems	R_s ($\Omega\cdot cm^2$)	R_{ct} ($\Omega\cdot cm^2$)	CPE ($\mu\Omega\cdot s^n\cdot cm^{-2}$)	n	C_{dl} ($\mu F\ cm^{-2}$)
CP	1.523	3.13	681.8	0.86239	255.6
CP-TMAB	1.821	4.389	670.9	0.75856	105.0
CP-TBAB	2.023	1.878	1315	0.68643	84.7
CP-BTAB	3.014	2.92	921.6	0.73843	113.3
CP-OTAB	2.116	2.13	666.4	0.85041	210.3
CP-DTAB	1.883	1.216	714.4	0.84156	189.5
CP-CTAB	1.983	0.54	800.2	0.84191	186.8
CP-HDBAB	1.382	2.631	767.6	0.83885	233.1
CP-EHDAB	1.784	1.1	673.6	0.86387	216.3

Supplementary Table 2. Simulated impedance parameters of Bode plots over CP electrode at different potentials.

E vs. Ag/AgCl (V)	R_s ($\Omega\cdot cm^2$)	R_{ct} ($\Omega\cdot cm^2$)	CPE ($\mu\Omega\cdot s^n\cdot cm^{-2}$)	n	C_{dl} ($\mu F\ cm^{-2}$)
–1.4	1.523	3.13	681.8	0.86239	255.6
–1.3	1.499	8.15	709.4	0.87224	333.5
–1.2	1.511	159.5	1033.6	0.81471	686.0
–1.1	1.457	355.2	1375.2	0.83486	1193.5
–1.0	1.503	426.3	675.4	0.82620	519.8
–0.9	1.468	500.7	667.3	0.82075	525.2
–0.8	1.448	534.7	622.5	0.82242	490.9

Supplementary Table 3. Simulated impedance parameters of Bode plots over CP-CTAB electrode at different potentials.

E vs. Ag/AgCl (V)	R_s ($\Omega\cdot cm^2$)	R_{ct} ($\Omega\cdot cm^2$)	CPE ($\mu\Omega\cdot s^n\cdot cm^{-2}$)	n	C_{dl} ($\mu F\ cm^{-2}$)
–1.4	1.983	0.54	800.2	0.84191	186.8
–1.3	2.035	1.097	623.9	0.86522	200.5
–1.2	1.998	10.77	963.8	0.82597	368.2
–1.1	2.000	154.8	1110.1	0.79922	713.2
–1.0	2.050	366.6	679.9	0.86269	545.0
–0.9	1.966	587.7	694.0	0.85708	597.6
–0.8	1.976	606.8	680.5	0.85655	586.8

Supplementary Table 4. Simulated impedance parameters of Bode plots over CP-DTAB electrode at different potentials.

E vs. Ag/AgCl (V)	R_s ($\Omega\cdot cm^2$)	R_{ct} ($\Omega\cdot cm^2$)	CPE ($\mu\Omega\cdot s^n\cdot cm^{-2}$)	n	C_{dl} ($\mu F\ cm^{-2}$)
–1.4	1.883	1.216	1014.4	0.84156	287.4
–1.3	1.840	1.688	1043.7	0.82767	278.7
–1.2	1.848	29.11	806.9	0.86340	445.7
–1.1	1.911	211.4	777.7	0.88536	615.6
–1.0	1.896	538.7	579.1	0.88914	500.8
–0.9	1.939	849.3	597.7	0.87403	542.1
–0.8	1.945	833.5	569.6	0.87153	510.4

Supplementary Table 5. Simulated impedance parameters of Bode plots over CP-HDBAB electrode at different potentials.

E vs. Ag/AgCl (V)	R_s ($\Omega\cdot cm^2$)	R_{ct} ($\Omega\cdot cm^2$)	CPE ($\mu\Omega\cdot s^n\cdot cm^{-2}$)	n	C_{dl} ($\mu F\ cm^{-2}$)
–1.4	1.382	2.631	767.6	0.83885	233.1
–1.3	1.245	4.802	918.4	0.86650	398.2
–1.2	1.393	5.868	684.1	0.87286	306.2
–1.1	1.389	200.5	589.2	0.88272	443.6
–1.0	1.369	535.6	679.1	0.88914	598.6
–0.9	1.364	660.7	651.9	0.88199	582.4
–0.8	1.356	728.7	661.3	0.87219	594.2

Supplementary Table 6. Simulated impedance parameters of Bode plots over CP-TMAB electrode at different potentials.

E vs. Ag/AgCl (V)	R_s (Ω·cm^2)	R_{ct} (Ω·cm^2)	CPE (μΩ·s^n·cm^{-2})	n	C_{dl} (μF cm^{-2})
–1.4	1.821	4.389	670.9	0.75856	105.0
–1.3	1.783	12.52	813.0	0.75051	176.9
–1.2	1.751	225.2	696.2	0.76562	394.8
–1.1	1.826	574.4	722.1	0.75877	545.9
–1.0	1.793	690.1	719.1	0.78043	590.4
–0.9	1.821	751.2	638.7	0.82708	547.8
–0.8	1.799	846.5	679.0	0.80795	595.3

Supplementary Table 7. Simulated impedance parameters of Bode plots over CP-TBAB electrode at different potentials.

E vs. Ag/AgCl (V)	R_s ($\Omega\cdot cm^2$)	R_{ct} ($\Omega\cdot cm^2$)	CPE ($\mu\Omega\cdot s^n\cdot cm^{-2}$)	n	C_{dl} ($\mu F\ cm^{-2}$)
–1.4	2.023	1.878	1315.0	0.68643	84.7
–1.3	1.999	4.491	1238.4	0.67631	103.2
–1.2	2.008	21.54	840.5	0.72310	180.9
–1.1	1.948	240.8	666.7	0.75419	367.3
–1.0	1.904	359.5	796.3	0.79086	572.0
–0.9	1.956	419.4	705.3	0.82150	541.3
–0.8	1.921	475.0	719.7	0.81017	559.7

Supplementary Table 8. Simulated impedance parameters of Nyquist plots at different temperatures at different reaction conditions.

T (K)	R_{ct} of each system ($\Omega \cdot cm^2$)				
	−1.4 V CP	−1.3 V CP	−1.4 V CP-CTAB	−1.3 V CP-CTAB	−1.4 V CP-TMAB
298	3.59	8.05	0.33	2.4	4.21
303	2.64	5.86	0.36	1.72	2.86
308	1.64	3.41	0.41	1.32	1.51
313	1.25	2.15	0.48	1.0	0.94
318	0.81	1.63	0.60	0.69	0.62

4. Supplementary References

1. Pan, B., Wang, Y. & Li, Y. Understanding and leveraging the effect of cations in the electrical double layer for electrochemical CO_2 reduction. *Chem Catal.* **2**, 1-10 (2022).
2. Hopkins, A.J., McFearin, C.L. & Richmond, G.L. SAMs under Water: The Impact of Ions on the Behavior of Water at Soft Hydrophobic Surfaces. *J. Phys. Chem. C* **115**, 11192-11203 (2011).
3. Jackson, M.N., Kaminsky, C.J., Oh, S., Melville, J.F. & Surendranath, Y. Graphite Conjugation Eliminates Redox Intermediates in Molecular Electrocatalysis. *J. Am. Chem. Soc.* **141**, 14160-14167 (2019).
4. Zuman, P. Aspects of Electrochemical Behavior of Aldehydes and Ketones in Protic Media. *Electroanalysis* **18**, 131-140 (2006).
5. Ünlü, M. et al. Analysis of Double Layer and Adsorption Effects at the Alkaline Polymer Electrolyte-Electrode Interface. *J. Electro. Soc.* **158** (2011).
6. Ong, A.L., Inglis, K.K., Whelligan, D.K., Murphy, S. & Varcoe, J.R. Effect of cationic molecules on the oxygen reduction reaction on fuel cell grade Pt/C (20 wt%) catalyst in potassium hydroxide (aq, 1 mol dm^{-3}). *Phys. Chem. Chem. Phys.* **17**, 12135-12145 (2015).
7. Yim, S.-D. et al. A Microelectrode Study of Interfacial Reactions at the Platinum-Alkaline Polymer Interface. *J. Electro. Soc.* **162**, F499-F506 (2015).
8. Ovalle, V.J., Hsu, Y.-S., Agrawal, N., Janik, M.J. & Waegele, M.M. Correlating hydration free energy and specific adsorption of alkali metal cations during CO_2 electroreduction on Au. *Nat. Catal.* **5**, 624-632 (2022).
9. McCrum, I.T., Hickner, M.A. & Janik, M.J. Quaternary Ammonium Cation Specific Adsorption on Platinum Electrodes: A Combined Experimental and Density Functional Theory Study. *J. Electro. Soc.* **165**, F114-F121 (2018).
10. Hua, W. et al. Optimizing the p charge of S in p-block metal sulfides for sulfur reduction electrocatalysis. *Nat. Catal.* **6**, 174-184 (2023).
11. Peng, L. et al. A fundamental look at electrocatalytic sulfur reduction reaction. *Nat. Catal.* **3**, 762-770 (2020).
12. Shimada, T. et al. Origin of the 2450 cm^{-1} Raman bands in HOPG, single-wall and double-wall carbon nanotubes. *Carbon* **43**, 1049-1054 (2005).
13. Singh, A.K., Yasri, N., Karan, K. & Roberts, E.P.L. Electrocatalytic Activity of Functionalized Carbon Paper Electrodes and Their Correlation to the Fermi Level Derived from Raman Spectra. *ACS Appl. Energy Mater.* **2**, 2324-2336 (2019).
14. Han, G., Liu, X., Cao, Z. & Sun, Y. Photocatalytic Pinacol C–C Coupling and Jet Fuel Precursor Production on ZnIn2S4 Nanosheets. *ACS Catal.* **10**, 9346-9355 (2020).
15. Reuben D. Rieke, S.-H.K. New Reagent for Reductive Coupling of Carbonyl and Imine Compounds: Highly Reactive Manganese-Mediated Pinacol Coupling of Aryl Aldehydes, Aryl Ketones, and Aldimines. *J. Org. Chem.* **63**, 5235-5239 (1998).

16. Zhong, Y. et al. An Artificial Electrode–Electrolyte Interface for CO_2 Electroreduction by Cation Surfactant Self-Assembly. *Angew. Chem. Int. Ed.* **59**, 19095-19101 (2020).
17. Zhao, G. & Zhu, H. Cation-π Interactions in Graphene-Containing Systems for Water Treatment and Beyond. *Adv. Mater.* **32**, e1905756 (2020).
18. Han, C. et al. Electrocatalytic hydrogenation of alkenes with Pd/carbon nanotubes at an oil–water interface. *Nat. Catal.* **5**, 1110-1119 (2022).
19. Liu, C.B. et al. Selectivity Origin of Organic Electrosynthesis Controlled by Electrode Materials: A Case Study on Pinacols. *ACS Catal.* **11**, 8958-8967 (2021).
20. Shin, S.J. et al. A unifying mechanism for cation effect modulating C_1 and C_2 productions from CO_2 electroreduction. *Nat. Commun.* **13**, 5482 (2022).
21. Li, J. et al. Hydroxide Is Not a Promoter of C_{2+} Product Formation in the Electrochemical Reduction of CO on Copper. *Angew. Chem. Int. Ed.* **59**, 4464-4469 (2020).
22. Dunwell, M., Luc, W., Yan, Y., Jiao, F. & Xu, B. Understanding Surface-Mediated Electrochemical Reactions: CO_2 Reduction and Beyond. *ACS Catal.* **8**, 8121-8129 (2018).
23. Pan, A., Kar, T., Rakshit, A.K. & Moulik, S.P. Enthalpy-Entropy Compensation (EEC) Effect: Decisive Role of Free Energy. *J. Phys. Chem. B* **120**, 10531-10539 (2016).
